# Sheep embryonic stem-like cells engrafted into sheep femoral condyle osteochondral defects: 4-year follow-up

**DOI:** 10.1186/s12917-018-1532-y

**Published:** 2018-06-28

**Authors:** Susanna Pilichi, Stefano Rocca, Maria Dattena, Roy Ransom Pool, Laura Mara, Daniela Sanna, Gerolamo Masala, Maria Lucia Manunta, Simone Dore, Andrea Manunta, Eraldo Sanna Passino

**Affiliations:** 1 Service of Research in Zootechnics, AGRIS Sardinia (Agricultural Research Agency of Sardinia), Olmedo, 07040 Sassari, Italy; 2 Department of Veterinary Pathobiology, College of Veterinary Medicine and Biomedical Sciences, Texas A&M University, College Station, TX 77843-4467 USA; 3 Department of Veterinary Medicine, via Vienna, 07100 Sassari, Italy; 4 National Reference Centre for Sheep and Goat Mastitis, Experimental Zooprophylactic Institute of Sardinia, via Duca degli Abruzzi 8, 07100 Sassari, Italy; 5 Department of Surgery, Microsurgery and Medicine, University of Sassari, viale San Pietro, 07100 Sassari, Italy

**Keywords:** Embryonic stem-like cells, Sheep, Articular cartilage, Teratoma

## Abstract

**Background:**

Articular cartilage lacks a regenerative response. Embryonic stem cells (ESCs) are a source of pluripotent cells for cartilage regeneration. Their use, however, is associated with a risk of teratoma development, which depends on multiple factors including the number of engrafted cells and their degree of histocompatibility with recipients, the immunosuppression of the host and the site of transplantation.

Colonies of sheep embryonic stem-like (ES-like) cells from in vitro-produced embryos, positive for stage-specific embryonic antigens (SSEAs), alkaline phosphatase (ALP), *Oct 4*, *Nanog*, *Sox 2* and *Stat 3* gene expression, and forming embryoid bodies, were pooled in groups of two-three, embedded in fibrin glue and engrafted into osteochondral defects in the left medial femoral condyles of 3 allogeneic ewes (ES). Empty defects (ED) and defects filled with cell-free glue (G) in the condyles of the controlateral stifle joint served as controls. After euthanasia at 4 years post-engraftment, the regenerated tissue was evaluated by macroscopic, histological and immunohistochemical (collagen type II) examinations and fluorescent in situ hybridization (FISH) assay to prove the ES-like cells origin of the regenerated tissue.

**Results:**

No teratoma occurred in any of the ES samples. No statistically significant macroscopic or histological differences were observed among the 3 treatment groups. FISH was positive in all the 3 ES samples.

**Conclusions:**

This in vivo preclinical study allowed a long-term evaluation of the occurrence of teratoma in non-immunosuppressed allogeneic adult sheep engrafted with allogeneic ES-like cells, supporting the safe and reliable application of ES cells in the clinic.

**Electronic supplementary material:**

The online version of this article (10.1186/s12917-018-1532-y) contains supplementary material, which is available to authorized users.

## Background

In synovial joints of humans and other long-lived species, articular cartilage withstands high levels of mechanical stress over many decades and continuously renews its extracellular matrix. However, it is vulnerable to injuries and diseases that cause irreparable tissue damage and lacks an effective regenerative response. Mechanical overload, caused by acute traumatic joint injury or obesity, or abnormalities in joint loading due to joint laxity or poor conformation, are the main causes of articular cartilage destruction and a risk factor for the development of secondary osteoarthritis [1, 2]. Indeed, they result in cell death (necrosis and/or apoptosis), disruption of the collagen matrix network, loss of cartilage proteoglycans, increased tissue water content and swelling, decreased mechanical functionality, up and down regulation of gene expression and impaired response to further mechanical stimulation [1]. A variety of procedures have been employed to restore damaged articular surfaces, including surgical treatments which, in most cases, result only in a delay in the onset of degenerative processes, and engraftment of cells, which is one of the most advanced technologies in cartilage regeneration. Embryonic stem cells (ESCs) are considered a promising source for tissue regeneration because of their ability to self-renew and pluripotency. However, their use raises several concerns related to immunologic incompatibility and possible development of teratoma [2–4].

The frequency of teratoma formation after engraftment varies in relation to the number of engrafted cells, the degree of histocompatibility between engrafted cells and recipient, the immunosuppressive treatment of the recipient and the site of transplantation, especially when it is an immune**-**privileged organ [4–7].

Theoretically, a single self-renewing undifferentiated ES cell should be sufficient to give raise to tumour [8]. Several studies focusing on allogeneic models of mouse ES cell transplantation have shown that as few as 400–500 mouse ES cells can lead to teratoma formation in immunodeficient mice and between 50,000 and 100,000 cells are required for tumour formation in immunocompetent animals [8].

To establish the influence of the degree of histocompatibility between engrafted cells and recipients, Dressel et al. [4] observed that subcutaneous injection of mouse ESCs (mESCs) in syngeneic hosts led to tumours in most cases, while they did not give rise to tumours in allogeneic and xenogeneic recipients, which differ in major antigens from syngeneic hosts. Thus, the immune system appears to be able to prevent tumour growth after injection of ESCs into allogeneic and xenogeneic hosts.

Before experimental engraftment of ESCs, immunosuppression is frequently used to avoid rejection of the engrafted cells, increasing the risk of teratoma formation. The effect of the immunosuppression on the tumorigenicity of the ESCs seems to be controlled by the natural killer (NK) cells, whose activity is not suppressed by the commonly used immunosuppressor agents [4].

An appropriate microenvironment is one of the main factors influencing the occurrence of tumours. In an in vivo study, Wakitani et al. [3] report that when ESCs are injected into the stifle joint they form teratomas, while when they are engrafted into articular cartilage defects they form cartilage [9].

In this context, the aim of this study was to evaluate the occurrence of teratoma formation in a long-term engraftment of allogeneic sheep ES-like cells into osteochondral defects of non-immunosuppressed adult sheep.

## Results

### In vitro embryo production (IVP) and sexing, isolation of ES-like cells, culture and characterization

As previously described [10], 80% of vitrified embryos successfully expanded after warming and about 50% were found to be male by sexing PCR, with 2 bands corresponding to the sex-determining region Y-linked gene (SRY) and to the sheep SAT 1114 DNA repeat unit (SAT) sequences, while female embryos showed only the band corresponding to the autosomal sequence SAT [10].

After 5–6 days of culture, about 35% of colonies were obtained [11], which formed monolayers of epithelioid cells with large clear nuclei, several prominent nucleoli, little cytoplasm and without trophectoderm-like or endoderm-like cells. By day 6, the colonies had overgrown to form cystic structures. Karyotype analysis of cells showed a normal number of chromosomes. All the tested colonies demonstrated their undifferentiated state by positive immunostaining with monoclonal antibodies (mAbs) against the surface antigens (Ag) SSEA-1, SSEA-3 and SSEA-4, by positive alkaline phosphatase staining [11] and positive gene expression for *Oct 4*, *Nanog*, *Sox 2* and *Stat 3* genes [12, 13]. Their undifferentiated state was confirmed by the absence of immunostaining with any of the Ag specific for differentiation [11].

Pluripotency was assessed by formation of embryoid bodies, which differentiated into tissues derived from the 3 embryonic germ layers, as confirmed by the positive immunostaining for the Ag for differentiation and negative immunostaining for SSEAs and alkaline phosphatase [11].

### Clinical assessment

All sheep walked normally by day 9 post-surgery. No further problems with locomotion were noted in any of the animals during the remainder of the study.

### Macroscopic assessment

**Fig. 1:**
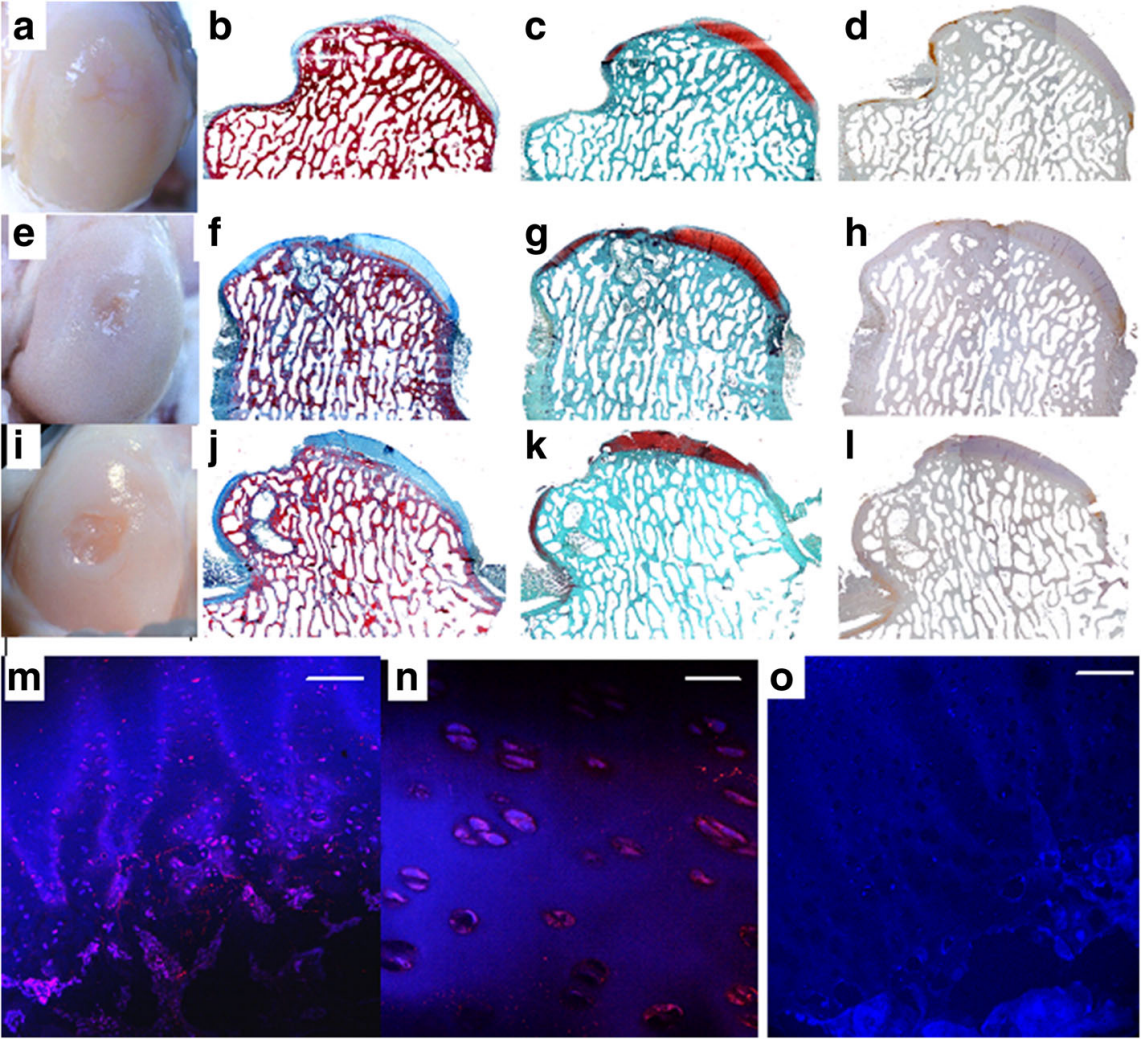
Embryonic stem-like cells engrafted in sheep femoral condyle osteochondral defects (ES), empty defect (ED) and only glue (G). **a**-**d**) ES at 4 years from surgery. **e**-**h**) ED at 4 years from surgery. I-L) G at 4 years from surgery. **a**-**e**-**i**) macroscopic appearance. **b**-**c**-**d**-**f**-**g**-**h**-**j**-**k**-**l**) histological sections, 1X magnification, bar: 1 mm. **b**-**f**-**j**) Azan-Mallory staining. **c**-**g**-**k**) Safranine-O staining. **d**-**h**-**l**) Collagen type II immunostaining. Fluorescent in situ hybridization (FISH): M) positive signals in chondrocytes derived from ES-like cells. 40X magnification; bar: 60 μm. N) Same field, 60X magnification; bar: 40 μm. O) Normal female adult articular cartilage from the right lateral femoral condyle (negative control). No signals are detected within chondrocytes. 20X magnification; bar: 120 μm

No tumour formation was observed in any of the ES samples (Fig. 1a).

No statistically significant macroscopic differences (*P* > 0.05) were observed among the 3 treatments (ES, ED and G) at 4 years post-surgery (Table. 1).

**Table 1:** Median (IQR)^d^ of macroscopic assessment among ES, ED and G at 4 years post-engraftment

Categories	ES^a^	ED ^b^	G ^c^	*P*-value
surface	1.5 (0.5–3.0)	1.0 (0.5–2.0)	1.0 (0.5–2.0)	0.86
filling	2.0 (0.0–3.0)	0.5 (0.5–2.0)	1.5 (1.5–2.5)	0.66
integration	2.5 (0.5–3.0)	1.5 (0.5–2.0)	1.0 (0.5–2.5)	0.66
total score	6.0 (1.0–9.0)	4.0 (1.5–5.0)	3.0 (3.0–7.0)	0.84

^a^ embryonic stem-like cells engrafted in the osteochondral defect created in the left medial femoral condyle; ^b^ empty defect created in the right medial condyle and left untreated; ^c^: glue filling in the osteochondral defect created in the right lateral condyle

^d^IQR: inter-quartile range

ES samples, however, showed a tendency toward a better healing with respect to both controls, for all categories (surface, filling and integration) (Table. 1) (Fig. 1a, e and i).

### Histological and immunohistochemical assessment

No signs of teratoma development were detected in any of the ES samples (Fig. 1b-d).

No statistically significant histological differences (P > 0.05) were observed among the 3 treatments (ES, ED and G) at 4 years post-surgery (Table. 2).

**Table 2:** Median (IQR)^d^ of histological assessment among ES, ED and G at 4 years post-engraftment

Categories	ES ^a^	ED ^b^	G ^c^	*P*-value
Filling od defect	2.0 (1.0–2.0)	1.5 (1.0–2.0)	1.0 (0.5–1.0)	0.19
Cartilage	10.0 (6.5–10.0)	8.5 (5.5–11.0)	7.5 (6.0–8.5)	0.62
Bone	7.0 (5.0–7.5)	5.5 (4.5–6.0)	6.0 (5.5–6.5)	0.39
Edges	1.5 (0.0–4.0)	0.5 (0.0–2.0)	1.0 (0.0–1.0)	0.73
Vascularity	6.0 (4.0–8.0)	4.5 (4.5–5.5)	3.5 (2.0–5.0)	0.25
Degeneration	7.5 (7.0–8.0)	7.0 (5.0–7.5)	7.0 (6.5–8.0)	0.53
Matrix staining	6.5 (1.5–8.5)	6.0 (4.0–8.0)	2.5 (1.5–4.5)	0.36
total score	45.5 (28.0–47.5)	35.5 (30.0–41.0)	33.5 (26.0–34.0)	0.39

^a^embryonic stem-like cells engrafted in the osteochondral defect created in the left medial femoral condyle; ^b^ empty defect created in the right medial condyle and left untreated; ^c^: glue filling in the osteochondral defect created in the right lateral condyle

^d^IQR: inter-quartile range

Once again, ES samples showed a tendency toward a better healing with respect to both controls for all examined categories, suggesting a beneficial effect on regeneration exerted by the treatment (Table. 2) (Fig. 1b-d, f-h, j-l).

All samples showed both immature hyaline cartilage, fibrocartilage and fibrous tissue at the upper part of the defect (with the exception of one G sample which was empty), and woven bone and subchondral ossification at the bottom. Integration of edges was complete only in one ES sample. Subchondral bone cysts were detected in 2 control samples: severe in 1 ED and moderate in 1 G sample (Fig. 1b-d, f-h, j-l).

### Fluorescent in situ hybridization (FISH) detection

The specificity of the chosen probe was confirmed by a Dot-Blot test. DNA derived from both ES samples and male fibroblasts were positive, and DNA from female fibroblasts was negative. FISH showed positive intranuclear signals only in the ES-like derived cells found in the newly formed tissue, while controls were negative (Fig. 1m-o).

## Discussion

To our knowledge, this is the first report describing the appearance of tissue arising from ES-like cells 4 years after placement in sheep femoral condyle osteochondral defects. Up to now, similar engraftments have been assessed at a maximum of 2 years post-surgery in sheep [14]; in most of the studies [15–18], animals were euthanized at 6 months, mimicking the end point used in the experiments performed in laboratory animals. In the authors’ opinion, 6 months is not sufficient to observe regeneration in large animals; other investigators have also commented on this [19]. Moreover, great methodological differences exist in experiments performed on sheep, with variable depth of defects [17, 20–23], surgical techniques [15, 24, 25] methods of cell delivery and variety of scaffolds [26, 27]. This great variability makes it very difficult, if not impossible, to compare results.

In this experiment, sheep were used since preclinical safety assessment of the potential for tumour formation in stem cell therapy requires animal studies of substantially longer duration than would be possible with small laboratory animals [2, 28, 29]. However, one limit of this study is the small number of sheep used. The 4-year post-surgery data reported here represents a part of a larger study where the healing of the defects was examined at shorter intervals [10, 14], and the purpose of this long-survival group was to look for development of teratomas. The study is clearly underpowered based on the few statistical units recruited per single arm. The initial null hypothesis cannot be rejected based on the non-significant results. The inference to a general population cannot be computed; however, this study can be the basis for the generation of further studies and hypotheses, whose sample size could be computed on the results we showed. Since no statistically significant differences were observed among the 3 treatments (ES, ED and G) at 4 years post-surgery, the previously reported histological scores at 2 years post-surgery [14] were compared with the current 4 year data (Additional file 2: Tables S3 and S4). While ES samples 4 years post-engraftment were similar to those at 2 years, both controls showed a delay in the bone regeneration process, with mainly woven bone and subchondral ossification in the samples at 4 years and lamellar and woven bone at 2 years. This slowing down could be due to individual variation, considering the small number of animals. The finding of moderate to severe subchondral bone cysts in the control samples in both time periods suggest that ES-like cells may confer a higher resistance to degeneration over the long term, as well as promote better integration of graft edges into the pre-existing cartilage. Poor integration of edges leaves a route for penetration of synovial fluid to the subchondral bone, with consequent bone resorption and cyst formation [15, 25, 30]. The beneficial effect exerted by ES-like cells could be due to their trophic action on the surrounding tissues by means of paracrine effects of growth factors, cytokines, chemokines, bioactive lipids, and extracellular micro-vesicles, which are released from the cells and have trophic, anti-apoptotic, and angiopoietic effects [31, 32].

Another limit was represented by the engraftment of the G control to the lateral femoral condyle. Medial and lateral condyles differ in shape, curvature, size, cartilage thickness [33] and contact forces [34]. The authors are aware that these differences can have affected the results. However, the alternative was to employ a different animal, increasing the individual variability and, thus, it was decided to use the lateral condyle of the same ewe.

No teratoma was detected in any of the 3 ES samples, either grossly or microscopically. This finding could be due to the particular experimental conditions employed, in accordance with others [7]. Cells were manipulated very little, derived from a pool of embryos and were engrafted in a low numbers. Moreover, the recipients were not immunosuppressed and the engraftment was placed in an immune-privileged site.

The culture conditions were determined by the behaviour of ungulate ES-like cells, which undergo early differentiation in vitro [35, 36]. To partially compensate for this, ES-like cells were collected at the first passage with little manipulation, thus preserving their pluripotency, as assessed by embryoid bodies formation [11]. With this procedure, it is possible to isolate, culture and characterize embryonic stem cell lines until the second passage without differentiation, as confirmed by the immunocytochemical, ALP and gene expression assays [11–13]. However, pluripotency is strictly linked to tumorigenicity, retaining similar gene expression profile, patterns of surface antigens, ability to self-renew and lack of contact inhibition [7, 37–39]. Nevertheless, tumorigenic cells show a limited capacity for differentiation and karyotype abnormalities, similarly to ESCs maintained for prolonged passages in culture [37].

Indeed, the collection at the first passage also avoids pathological miss-differentiation, which might occur in some cells during numerous in vitro passages, as claimed by some authors [4]. The common hypothesis is that only undifferentiated stem cells can give rise to teratoma and that teratoma formation after injection of differentiated cells is caused by contamination of the grafts with undifferentiated cells. Dressel, however, established that when both ESCs and in vitro differentiated cells derived from ESCs were injected into hosts, teratoma was equally frequent, despite the differentiated cell population contained less than 5% of undifferentiated cells [4]. This finding clearly indicates that differentiated cells must contain a tumorigenic cell population that is different from ESCs and which might be even more tumorigenic than ESCs. These cells might represent a physiological differentiation step or, alternatively, a pathological miss-differentiation [4], or they may retain chromosomal aberrations [40, 41] due to adaptations that occur during the in vitro expansion through high numbers of passages in culture and in vitro differentiation.

To avoid genetic manipulations, the sex-determining region Y-linked gene sequence (SRY) was chosen as a marker for proof of the origin of the newly formed tissue from ES-like cells. The method was demonstrated to be effective and reliable. Several authors employ green fluorescent protein (GFP) gene as a marker in engrafted cells [18, 42, 43]. However, cell fusion might occur that could lead to the expression of this marker gene in hybrid cells [44], and transfection of ESCs with GFP gene could alter the ESCs’ immunogenicity [28, 45, 46].

There is a great difference of opinion on the optimal number of ESCs to implant. The very small number of cells (5 × 10^5^) used in this experiment may have been insufficient for teratoma development in sheep [10]. In general, it has been established that the minimum number of ESCs capable of forming teratomas depends on the site of delivery, the method of cell administration, the immunological behaviour and the species, and ranges from 2/4-5 × 10^2^ for mESCs to 1 × 10^4^/1 × 10^6^ for human ESCs (hESCs) [7, 8, 44].

Thus, it is probable that other factors affected the absence of teratoma development, such as the degree of histocompatibility. In a syngeneic setting, the immune system appears to be able to reject small numbers of pluripotent stem cells or to decelerate teratoma growth after injection of higher number of cells [46]. In allogeneic models, the degree of histocompatibility appears to be one important factor that determines the outcome of a transplantation [44].

In our allogeneic setting, no signs of inflammation were detected, in agreement with results from a previous study [14] where inflammatory cells were not detected at 1 month post-surgery. Commonly used immunosuppressant treatments do not inactivate the NK cells, whose cytotoxic activity is able to prevent the growth of tumour derived from ESCs but not from differentiated cells [4]. This activity is controlled by a set of inhibitory and activating receptors expressed on NK cells, which interact with certain ligands on target cells. Ligands of inhibitory NK receptors are Major Histocompatibility Complex (MHC) class I molecules, which are not detectable on ESCs but are slightly up-regulated during differentiation [4, 7]. This could explain the susceptibility of ESCs to cytotoxic activity of NK cells, together with other immune mechanisms, such as the complement system, or the degree of inflammatory response after engraftment, with the attraction of Interleukin 2, which activates the NK cells. On the contrary, activating ligands are expressed in ESCs, and they might signal the presence of potentially dangerous cells to the immune system, triggering the mechanism of the immune surveillance, involved in the detection of tumour cells [4].

In this experiment, non-immunosuppressed sheep were used for the engraftment of allogeneic ES-like cells. Thus, it might be expected that the host’s immune system would reject the engraftment. However, engrafted ES-like cells were derived from a pool of colonies arising from at least two or three embryos, and therefore presented heterogeneous sets of antigens, maybe insufficient to stimulate the host response but perhaps able to develop a teratoma. This is in agreement with others [7, 47], who affirm that, among the several strategies to minimize rejection of ESCs transplants, there is the chimerism. In addition, immunosuppression, rather than increase the risk of tumour formation derived from stem cells graft [44], can be used in animals but may not be acceptable in human trials and immunotoxicity of the administered drug is difficult to assess in immunosuppressed animals [28].

The local signals from the tissue into which cells are engrafted, including direct cellular interactions with adjacent host tissues, exposure to secreted morphogens, and more general homeostatic signals, influence the ability of the grafted cells to differentiate [7, 8, 39, 43]. Thus, under these experimental conditions, osteochondral defects may have forced cells to differentiate towards the chondrogenic lineage, as previously reported [14]. Several studies have demonstrated that the biological environment of tissues such as bone and cartilage is highly influenced by mechanical forces felt by the cells [27, 43]. Mechanical stress acts in osteochondral defects, confining and pressing down the engrafted cells so that they cannot produce a large mass that grows out of the defect, as assessed by Nakajima et al. [43], who report the development of teratoma after engraftment of 1 × 10^7^ ESCs in osteochondral defects of immobilized rats, but not in joint free animals. Moreover, Wakitani et al. observed that ESCs produced a greater ratio of cartilaginous tissues in teratomas grown in the knee joint compared with cells grafted into the subcutaneous space, suggesting that implanted cells tend to differentiate into tissues resembling their surroundings [6].

Articular cartilage is considered an immune privileged site, partly due to the lack of vasculature and the dense extracellular matrix of this tissue, where no or slow allograft rejection is observed, due to the limited access of the host’s immunocompetent cells [43]. Although this might increase the risk of teratoma formation [44, 46], it could be prevented by exposure of articular cartilage to the immune system through the synovial fluid that bathes its surface and subchondral bone, especially if it has suffered injury. NK cells can migrate into the engraftment via blood circulation following release of chemotactic factors from inflammatory cells [46].

To better understand the tumorigenic potential of ES-like cells employed in this experiment, they were injected into a subcutaneous space in the inner thigh of 6 sheep, which were euthanized at 2, 4 and 6 months from surgery. These time-periods was considered sufficient to assess the teratoma development, as confirmed by others [3, 4, 8, 9, 39]. Probably the immune system reabsorbed the cells, since no signs of immune rejection or teratoma formation were detected, both at macroscopic and histological level. This finding is in agreement with others [7, 48], who reported that hESCs are completely eliminated at 1 month post-transplantation, but in contrast with Li et al. [49], who established that hESCs failed to elicit immune response during the first 48 h post-transplantation.

Assessment of the risk of tumour formation by pluripotent stem cell-based therapies is an essential part of safety evaluation. Biological assays that enable the detection of a malignant phenotype in pluripotent stem cells are of enormous value, since the appearance of such features in ESC-derived teratomas would suggest the presence of transformed cells in the cultures [29]. Gropp et al. [50] established the key points for a teratoma assay that is sensitive, quantitative, and easy to perform and to monitor. Among them, there are the identification of the right recipient animals, and the duration of monitoring for tumour formation. In vivo assays performed on laboratory animals are hampered by the relatively short life span of rodents, which precludes long-term monitoring of grafts that may persist in patients for decades [29]. On the contrary, this goal can be achieved in large animals.

## Conclusions

While stem cell therapy is rapidly advancing, the science of stem cell safety assessment must also evolve. It is largely accepted that safety analysis should include evaluation of the tumorigenic potential of the cells after transplantation into the site of engraftment, using methods of delivery that mimic, as best as possible, the planned clinical scenario. Strong support from data obtained in pre-clinical models is mandatory in the development of a new cell therapy. Under this point of view, the authors believe that the present study can add a useful note to the safe and reliable clinical translation of ES cells. Further investigations are necessary to standardize a protocol for the production of “real” sheep ESCs and to study in more depth the closely linked fields of tumorigenicity, pluripotency and immunogenicity.

## Methods

Except where otherwise indicated, all chemicals were obtained from Sigma-Aldrich (Milan, Italy).

### Study design

Three Sarda ewes, about 4.5 years old and weighing approximately 45 kg, without muscular-skeletal pathologies, were used in this experiment. ES-like cells were engrafted in the osteochondral defect created in the left medial femoral condyle (ES), while 2 identical defects were created in the condyles of the controlateral stifle joint: the medial condyle was left untreated (empty defect: ED), while the lateral condyle was filled with glue (G), and both served as controls. Thus, a total of 9 defects were created: 3 ES, 3 ED and 3 G. To eliminate all the variables among animals, each animal served as its own control. Sheep were euthanized at 4 years post-surgery.

### In vitro embryo production (IVP) and sexing, isolation of ES-like cells, culture, and characterization

Sheep embryos were produced, vitrified and warmed following in vitro procedures as described by Dattena et al. [51]. Embryos underwent immunosurgical complement mediated lysis of trophoblastic cells to isolate the inner cell mass (ICM) before being cultured according to Dattena et al. [11]. The *O. aries* Y chromosome repeat region OY 11.1 DNA sequence (SRY; sex determining region Y-linked gene sequence) [GenBank: U30307] was used to detect ES-like cells in the regenerated tissue, thus only male embryos were used to produce the engrafted cells which were selected by means of a duplex PCR performed on trophoblastic cells released during immunosurgery, as previously described [10]. Briefly, two sets of primers were used: the first recognized the SRY sequence (primers sequence: forward, 5-CTCAGCAAAGCACACCAGAC-3; reverse, 5-GAACTTTCAAGCAGCTGAGGC-3) and produced a 301 base pair (bp) fragment in male samples, while the latter, used as a positive control, recognized the autosomal sequence sheep 1.714 satellite DNA repeat unit [GenBank: X01839] (primers sequence: forward, 5-AGGTGTTCTCGACTTACGAT-3; reverse, 5-CTCGAGAGGAGAACTGACTC-3) and yielded a 216 bp fragment in both males and females. Amplification products (15 μl) were analysed on 2% agarose gel (Applied Biosystems Thermo Fisher), stained with ethidium bromide and evaluated under ultraviolet light. If one band (216 bp), corresponding to the autosomal sequence, was visible on the gel, the sample was considered female, whereas the presence of two bands (216 and 301 bp), corresponding to the satellite and Y chromosome sequences, indicated that the sample was derived from a male.

ES-like colonies were characterized according to Dattena et al. [11]. Briefly, surface antigens for staminality SSEA-1, SSEA-3 and SSEA-4 (Developmental Studies Hybridoma Bank - DSHB, University of Iowa, Iowa City, IA) were detected immunocytochemically, while the expression of the *Oct4*, *Nanog*, *Sox2* and *Stat3* genes, up-regulated during the embryo development, was assessed by the gene expression technology [12, 13]. To assess the absence of differentiation towards the germinal layers, early mesoderm (FE-C6), embryonic myosin (F1–652), neural precursor cell (FORSE-1), and endoderm (anti-cytokeratin 18) specific primary mAbs (DSHB, University of Iowa, Iowa City, IA) were used. All results were observed under an inverted fluorescence and phase contrast microscope (Nikon Diaphot 200) coupled to a digital camera for imaging capture (DFC450 C, Leica) and to a software for image acquisition (Leica Application Suite - LAS).

### Graft preparation and surgical phase

Two or three male ES-like colonies were harvested and pooled together before disaggregating, as previously described [11]. About 5 × 10^5^ cells were embedded in 60 μl fibrin glue to be engrafted into the cartilage defect.

All surgical procedures were performed as previously described [14]. The osteochondral defect was 6 mm wide and 2 mm deep, involving the subchondral bone. A motorized drill for spinal surgery with a stop-mechanism was used, so that the depth drilled was exactly the same in all samples. The medial condyle of the left joint received the ES-like cells embedded in fibrin glue (ES), while in the right knee, the medial condylar defect was left untreated (ED) and the lateral condylar defect was filled with fibrin glue without added ES-like cells (G). Animals were housed in a small enclosure for the initial 10 post-surgical days to restrict movement and were then returned to pasture. Euthanasia was performed at 4 years after surgery, under veterinary supervision: after sedation (Diazepam 0.4 mg/kg IV), anesthesia was induced by injection of Thiopental 20 mg/kg IV, followed by sacrifice by means of Tanax® 10 ml IV.

### Macroscopic assessment

The regenerated tissue in the cartilage defect was examined grossly by two blinded observers, using a semi-quantitative scoring system, developed by the International Cartilage Repair Society (ICRSS) (Additional file 1: Table S1), and the values obtained were averaged. One possible limit of this scoring system is the absence of the assessment of transverse sections, perpendicular to the articular surface, which could have allowed evaluation of the quality of the newly formed tissue deep in the samples, together with the detection of subchondral bone cysts in the gross specimens.

### Histological and immunohistochemical assessment

The articular defects in the condyles were removed as osteochondral blocks about 20 mm in width and 10–15 mm deep and processed for histochemistry and immunohistochemistry for collagen type II, as previously described [14]. Briefly, sections were stained with hematoxylin and eosin to evaluate the general tissue morphology, Azan-Mallory and collagen type II to detect the collagen arrangement and type, and Safranine-O to observe the proteoglycan distribution in the cartilage matrix. Image results were captured under a light microscope (Nikon Eclipse 80i) connected to a digital camera for imaging capture (Nikon DS-L2 camera control unit) and the Nikon dedicated software 3422.1001.1798.080117 (Nikon Instruments Inc., Melville, NY).

The histological and immunohistochemical evaluation was performed by 2 independent observers using a grading system developed by the authors and derived from Caplan [52] (Additional file 1: Table S2), and the 2 values obtained for each sample were averaged. A total score of 5 indicated the worst possible healing, while 56 indicated the best.

### Fluorescent in situ hybridization (FISH) detection

To detect ES-like cells in the regenerating tissue, the *O. aries* Y chromosome repeat region OY 11.1 DNA sequence [GenBank: U30307] was used and its presence was assessed by FISH, as previously described [14], using the DNA probe 50 bp in length (biotin-5’AAAGGGAGGGAGAGACCAAAGAAGTAGATGATGATGATGATGAAGTGATC 3′) built on the SRY sequence. Images were captured under a confocal microscope (Leica Microsystems, Germany) and processed using LAS AF Lite application software, developed by Leica Microsystems CMS GmbH for contrast and brightness adjustment. To assess the effectiveness of the probe, a membrane Dot-Blot test had been previously performed on the ES-like cell samples, using male and female fibroblasts as positive and negative controls, respectively.

### Statistical analysis

Normality distribution of the collected variables was assessed with the Shapiro-Wilk normality test. Median and inter-quartile range (IQR) were used to describe quantitative variables. Statistical differences between macroscopic and histological scores of the 3 treatments (ES, ED and G) were evaluated performing the Kruskall-Wallis analysis, adjusting statistical significance for the multiple comparisons (Bonferroni correction). A two-sided *p*-value of < 0.05 was used to consider a difference statistically significant. Statistical analysis was carried out using STATA®12 (StataCorp, College Station, TX, USA).

## Additional files

Additional file 1: **Table S1.** Semi quantitative scoring key for macroscopic evaluation of regenerated cartilage defects at 4 years after surgery (9 = normal; 0 = worst healing). **Table S2.** Semi-quantitative score for histological evaluation of regenerated cartilage defects at 4 years after surgery (56 = maximum score; 5 = minimum score). (DOCX 22 kb)

Additional file 2: **Table S3.** Median (IQR)^*^ of comparison between macroscopic assessment of treatments (ES, ED and G) at 2 and 4 years. **Table S4.** Median (IQR)^*^ of comparison between histological assessment of treatments (ES, ED and G) at 2 and 4 years. (DOCX 18 kb)

## Abbreviations

Ag: Antigens
ALP: Alkaline phosphatase
ED: Empty defect
ES: ES-like cells engrafted in the medial femoral condyle defect
ESCs: Embryonic stem cells
ES-like cells: Embryonic stem-like cells
FISH: Fluorescent in situ
G: Glue
GFP: Green fluorescent protein
hESCs: Human ESCs
ICM: Inner cell mass
IQR: Inter-quartile range
IVP: In vitro embryo production
mAbs: Monoclonal antibodies
mESCs: Mouse ESCs
MHC: Major histocompatibility complex
NK: Natural killer cells
SAT: Sheep SAT 1114 DNA repeat unit sequence
SRY: Sex-determining region Y-linked gene sequence
SSEAs: Stage-specific embryonic antigens
