# Supplementary material for: Sheep embryonic stem-like cells engrafted into sheep femoral condyle osteochondral defects: 4-year follow-up
Source: BMC Vet Res. 2018 Jun 28;14:213. doi: 10.1186/s12917-018-1532-y (PMC6022494; doi:10.1186/s12917-018-1532-y)
Supplement: Supplementary file 1 — Table S1. Semi quantitative scoring key for macroscopic evaluation of regenerated cartilage defects at 4 years after surgery (9 = normal; 0 = worst healing). Table S2. Semi-quantitative score for histological evaluation of regenerated cartilage defects at 4 years after surgery (56 = maximum score; 5 = minimum score). (DOCX 22 kb) [file 12917_2018_1532_MOESM1_ESM.docx]

**Table S1**: Semi quantitative scoring key for macroscopic evaluation of regenerated cartilage defects at 4 years after surgery (9 = normal; 0 = worst healing).

| **categories** | **Score** | **Qualifications** |
| --- | --- | --- |
| Surface of texture of repair tissue | 3 | Normal: smooth, white* |
|  | 2 | >75% normal |
|  | 1 | 50-75% normal |
|  | 0 | <50% normal |
| Percent area of defect filled | 3 | 100% |
|  | 2 | >75% |
|  | 1 | 50-75% |
|  | 0 | <50% |
| Graft-recipient tissue integration | 3 | 100% of perimeter |
|  | 2 | >75% |
|  | 1 | 50-75% |
|  | 0 | <50% |

* Indistinguishable from normal healthy cartilage.

**Table S2:** Semi-quantitative score for histological evaluation of regenerated cartilage defects at 4 years after surgery (56 = maximum score; 5 = minimum score)

| **Categories** |  | **Qualifications** | **score** |
| --- | --- | --- | --- |
| Filling of defect |  | Complete | **2** |
|  |  | Partial | **1** |
|  |  | None | **0** |
| Cartilage | Surface regularity | Regular | **2** |
|  |  | Irregular | **1** |
|  | Cell type) | Chondroblasts/cytes | **4** |
|  |  | Both chondroblasts & fibroblasts | **3** |
|  |  | fibroblasts | **2** |
|  |  | fibroblasts & endothelial cells | **1** |
|  |  | Empty | **0** |
|  | Tissue type | Mature hyaline cartilage | **5** |
|  |  | Immature hyaline cartilage | **4** |
|  |  | Fibrocartilage | **3** |
|  |  | Fibrous tissue | **2** |
|  |  | Granulation tissue | **1** |
|  |  | Empty | **0** |
|  | Continuity tangential layer proliferation & subchondral ossification | Presence | **1** |
|  |  | Absence | **0** |
| Bone | Ossification | Lamellar bone | **3** |
|  |  | Woven bone | **2** |
|  |  | Subchondral ossification | **1** |
|  |  | Absence | **0** |
|  | Cell type | Osteogenic cells | **5** |
|  |  | Chondroblasts/cytes | **4** |
|  |  | Both chondroblasts & fibroblasts | **3** |
|  |  | fibroblasts | **2** |
|  |  | fibroblasts & endothelial cells | **1** |
|  |  | Empty | **0** |
| edges of defect | Continuity articular cartilage edge and filling material | 2 edges | **2** |
|  |  | 1 edge | **1** |
|  |  | None | **0** |
|  | Tidemark | Presence (complete) | **2** |
|  |  | Presence (intermitted) | **1** |
|  |  | Premature/absence | **0** |
| Vascularity | Amount | None | **3** |
|  |  | Low | **2** |
|  |  | Medium | **1** |
|  |  | High | **0** |
|  | Distribution | Absence | **5** |
|  |  | Zone I (above level of tidemark) | **4** |
|  |  | Zone II (upper half of defect below tidemark) | **3** |
|  |  | Zone III (lower half of defect below tidemark) | **2** |
|  |  | entire | **1** |
| Degeneration | Degree of articular deterioration | None | **4** |
|  |  | Grade I (Mild) | **3** |
|  |  | Grade II (Moderate) | **2** |
|  |  | Grade III (Severe) | **1** |
|  | Subchondral bone sclerosis | None or no pertinent | **4** |
|  |  | Grade I (Mild) | **3** |
|  |  | Grade II (Moderate) | **2** |
|  |  | Grade III (Severe) | **1** |
|  | Subchondral bone cysts | None | **4** |
|  |  | Grade I (Mild) | **3** |
|  |  | Grade II (Moderate) | **2** |
|  |  | Grade III (Severe) | **1** |
| Matrix staining | Collagen staining  (Azan-Mallory) | Grade I (marked intensity) | **3** |
|  |  | Grade II (moderate intensity) | **2** |
|  |  | Grade III (mild intensity) | **1** |
|  |  | Premature/negative | **0** |
|  | Proteoglycan staining (Safranin/O) | Grade I (marked intensity) | **4** |
|  |  | Grade II (moderate intensity) | **3** |
|  |  | Grade III (mild intensity) | **2** |
|  |  | Grade IV (initial production) | **1** |
|  |  | Premature/negative | **0** |
|  | Collagen type II | Grade I (marked intensity) | **3** |
|  |  | Grade II (moderate intensity) | **2** |
|  |  | Grade III (mild intensity) | **1** |
|  |  | Premature/negative | **0** |
