# Supplementary material for: Sheep embryonic stem-like cells engrafted into sheep femoral condyle osteochondral defects: 4-year follow-up
Source: BMC Vet Res. 2018 Jun 28;14:213. doi: 10.1186/s12917-018-1532-y (PMC6022494; doi:10.1186/s12917-018-1532-y)
Supplement: Supplementary file 2 — Table S3. Median (IQR)* of comparison between macroscopic assessment of treatments (ES, ED and G) at 2 and 4 years. Table S4. Median (IQR)* of comparison between histological assessment of treatments (ES, ED and G) at 2 and 4 years. (DOCX 18 kb) [file 12917_2018_1532_MOESM2_ESM.docx]

**Table S3**: Median (IQR)^*^ of comparison between macroscopic assessment of treatments (ES, ED and G) at 2 and 4 years

| **Categories** | **2 years** | **4 years** |  |
| --- | --- | --- | --- |
|  | **ES** ^a^ | **ES** | **P-value** ^d^ |
| surface | 2.0 (2.0-2.5) | 1.5 (0.5-3.0) | 0.39 |
| filling | 3.0 (2.5-3.0) | 2.0 (0.0-3.0) | 0.15 |
| integration | 2.0 (2.0-2.0) | 2.5 (0.5-3.0) | 0.34 |
| total score | 7.0 (6.0-7.5) | 6.0 (1.0-9.0) | 0.68 |
|  | **ED** ^b^ | **ED** | **P-value** |
| surface | 1.5 (0.5-2.5) | 1.0 (0.5-2.0) | 0.72 |
| filling | 2.0 (1.5-2.5) | 0.5 (0.5-2.0) | 0.14 |
| integration | 2.0 (1.5-2.0) | 1.5 (0.5-2.0) | 0.33 |
| total score | 5.5 (3.5-7.0) | 4.0 (1.5-5.0) | 0.21 |
|  | **G** ^c^ | **G** | **P-value** |
| surface | 1.0 (0.0-2.0) | 1.0 (0.5-2.0) | 0.82 |
| filling | 1.0 (1.0-2.0) | 1.5 (1.5-2.5) | 0.26 |
| integration | 0.0 (0.0-2.0) | 1.0 (0.5-2.5) | 0.27 |
| total score | 2.0 (1.0-6.0) | 3.0 (3.0-7.0) | 0.27 |

^a^: embryonic stem-like cells engrafted in the osteochondral defect created in the left medial femoral condyle; ^b^: empty defect created in the right medial condyle and left untreated; ^c^: glue filling in the osteochondral defect created in the right lateral condyle; ^d^: statistical differences between values collected at 2 and 4 years were calculated performing the Mann–Whitney U test.

^*^ IQR: inter-quartile range

**Table S4**: Median (IQR)^*^ of comparison between histological assessment of treatments (ES, ED and G) at 2 and 4 years.

| **Categories** | **2 years** | **4 years** |  |
| --- | --- | --- | --- |
|  | **ES** ^a^ | **ES** | **P-value** ^d^ |
| Filling of defect | 2.0 (1.0-2.0) | 2.0 (1.0-2.0) | 0.90 |
| cartilage | 10.0 (6.5-10.5) | 10.0 (6.5-10.0) | 0.83 |
| bone | 8.0 (7.0-8.0) | 7.0 (5.0-7.5) | 0.10 |
| edges | 3.0 (2.5-3.5) | 1.5 (0.0-4.0) | 0.46 |
| vascularity | 8.0 (6.0-8.0) | 6.0 (4.0-8.0) | 0.41 |
| degeneration | 8.5 (6.0-9.0) | 7.5 (7.0-8.0) | 0.53 |
| matrix staining | 9.5 (8.0-10.0) | 6.5 (1.5-8.5) | 0.09 |
| total score | 46.5 (40.5-50.0) | 45.5 (28.0-47.5) | 0.54 |
|  | **ED** ^b^ | **ED** | **P-value** |
| Filling of defect | 1.5 (1.0-2.0) | 1.5 (1.0-2.0) | 1.00 |
| cartilage | 7.5 (6.5-8.5) | 8.5 (5.5-11.0) | 0.72 |
| bone | 8.0 (7.5-8.0) | 5.5 (4.5-6.0) | 0.03 |
| edges | 1.5 (0.5-2.5) | 0.5 (0.0-2.0) | 0.47 |
| vascularity | 3.5 (3.0-4.0) | 4.5 (4.5-5.5) | 0.03 |
| degeneration | 7.0 (6.5-7.5) | 7.0 (5.0-7.5) | 0.71 |
| matrix staining | 7.0 (4.5-8.5) | 6.0 (4.0-8.0) | 0.72 |
| total score | 35.5 (31.5-39.0) | 35.5 (30.0-41.0) | 0.59 |
|  | **G** ^c^ | **G** | **P-value** |
| Filling of defect | 1.5 (0.5-2.0) | 1.0 (0.5-1.0) | 0.46 |
| cartilage | 7.5 (6.5-9.0) | 7.5 (6.0-8.5) | 0.86 |
| bone | 7.5 (7.0-8.0) | 6.0 (5.5-6.5) | 0.03 |
| edges | 2.0 (0.5-3.0) | 1.0 (0.0-1.0) | 0.35 |
| vascularity | 6.0 (3.5-8.0) | 3.5 (2.0-5.0) | 0.28 |
| degeneration | 7.0 (5.0-8.0) | 7.0 (6.5-8.0) | 0.71 |
| matrix staining | 8.0 (6.0-8.5) | 2.5 (1.5-4.5) | 0.07 |
| total score | 38.5 (29.0-46.5) | 33.5 (26.0-34.0) | 0.86 |

^a^: embryonic stem-like cells engrafted in the osteochondral defect created in the left medial femoral condyle; ^b^: empty defect created in the right medial condyle and left untreated; ^c^: glue filling in the osteochondral defect created in the right lateral condyle; ^d^: statistical differences between values collected at 2 and 4 years were calculated performing the Mann–Whitney U test.

^*^ IQR: inter-quartile range
